# Chronotherapy in Hematological Malignancies: Evaluating the Rationale and Evidence

**DOI:** 10.1002/prp2.70302

**Published:** 2026-07-31

**Authors:** Andrej Belančić, Marko Skelin, Yun Wah Lam, Almir Fajkić, Bruna Perkov‐Stipičin, Rossana Roncato, Ivan Krečak, Marko Lucijanić

**Affiliations:** ^1^ Department of Basic and Clinical Pharmacology and Toxicology University of Rijeka, Faculty of Medicine Rijeka Croatia; ^2^ General Hospital Šibenik Šibenik Croatia; ^3^ Department of Diagnostic Sciences, School of Nursing and Health Sciences Hong Kong Metropolitan University Hong Kong SAR China; ^4^ Department of Pathophysiology University of Sarajevo Sarajevo Bosnia and Herzegovina; ^5^ Department of Medicine University of Udine Udine Italy; ^6^ Faculty of Medicine University of Rijeka Rijeka Croatia; ^7^ University of Applied Sciences, Šibenik Croatia; ^8^ Division of Haematology, Department of Internal Medicine Clinical Hospital Dubrava Zagreb Croatia; ^9^ Department of Scientific Research and Translational Medicine Clinical Hospital Dubrava Zagreb Croatia; ^10^ Department of Internal Medicine School of Medicine University of Zagreb Zagreb Croatia

**Keywords:** chronopharmacology, chronotherapy, circadian rhythms, digital health, hematological malignancies, precision medicine, treatment timing

## Abstract

Hematological malignancies remain one of the leading causes of morbidity and mortality despite advances in targeted therapies, immunotherapy, and stem cell transplantation. Emerging evidence indicates that treatment efficacy and toxicity depend not only on the choice of therapy but also on its timing relative to the patient's internal circadian rhythm. The circadian clock orchestrates fundamental processes in hematopoiesis and immunity, such as stem‐cell proliferation, leukocyte trafficking, DNA repair, and drug metabolism, while its disruption promotes malignant transformation, therapeutic resistance, and systemic toxicity. This narrative review synthesizes current understanding of circadian regulation in hematopoietic and immune systems, the mechanistic and preclinical foundations of chronotherapy in blood cancers, and the limited but growing body of clinical evidence linking treatment timing with outcome in leukemia, lymphoma, and transplantation. The review also examines practical challenges, including inter‐individual variability, disease‐induced circadian disruption, and hospital workflow constraints, while highlighting emerging technologies, such as transcriptomic clocks, wearable biosensors, and AI‐driven scheduling algorithms, that are poised to enable personalized, time‐aware therapy. By integrating temporal precision into existing therapeutic frameworks, chronotherapy may represent a promising investigational dimension of precision medicine in hematological oncology. However, its clinical value remains to be defined through prospective studies that incorporate validated circadian biomarkers, predefined timing windows, and clinically meaningful efficacy and toxicity endpoints.

## 1 Introduction

Hematological malignancies, including leukemia, lymphoma, and myeloma, comprise a diverse group of cancers arising from the malignant transformation of hematopoietic and immune cells. Collectively, they account for around 7% of all cancers worldwide and remain a major cause of morbidity and mortality [1]. Despite major therapeutic progress through targeted agents, immunotherapies, and stem‐cell transplantation, many patients continue to experience relapse, treatment resistance, and therapy‐related toxicity. These challenges have driven the development of innovative treatment strategies, including the emerging discipline of temporal oncology, which seeks to integrate circadian biology into therapeutic design and clinical practice. Growing evidence now demonstrates that circadian biology can shape not only disease pathogenesis but also treatment response, suggesting that treatment outcomes may depend not only on what therapies are used, but also on when they are administered [2].

Circadian regulation coordinates 24‐h rhythms in gene expression, metabolism, immune activity, and cellular repair across nearly all tissues. These rhythms are synchronized by systemic cues such as light exposure, feeding‐fasting cycles, sleep–wake behavior, and hormonal fluctuations, while the molecular architecture of the clock is outlined in the following section [3, 4, 5, 6]. Within the hematopoietic system, circadian regulation shapes key physiological processes, including stem cell proliferation, immune cell trafficking, and cytokine signaling. Disruption of these rhythms through genetic, behavioral, or environmental factors can impair hematopoietic homeostasis, weaken immune surveillance, and contribute to malignant transformation [2, 4, 5]. Thus, the alignment between internal and external time is increasingly recognized as a determinant of physiological integrity, and its chronic disturbance, termed circadian misalignment, has emerged as a biological risk factor for both oncogenesis and tumor progression.

In solid tumors, a multitude of clinical trials has shown that synchronizing chemotherapy or immunotherapy with circadian phase can improve drug tolerability and, in some cases, therapeutic outcomes [7, 8, 9]. These time‐dependent effects reflect rhythmic variations in cellular proliferation, DNA repair, hormone secretion, and drug metabolism. Experimental models and small observational studies suggest that circadian rhythms influence not only cancer biology but also drug metabolism, DNA repair, and tissue recovery [2]. These findings imply that time of administration may represent a critical yet underexplored dimension of therapeutic precision in blood cancers. This therapeutic principle, known as chronotherapy, refers to the deliberate scheduling of treatment according to the body's circadian rhythms to maximize efficacy while minimizing toxicity.

Despite a strong biological rationale, chronotherapy has not yet been systematically incorporated into hematological oncology. Barriers include the absence of validated circadian biomarkers, the inter‐individual variability of sleep–wake and hormonal cycles, and the logistical challenges of integrating time‐specific dosing into standard hospital workflows. However, technological advances, such as wearable sensors, blood‐based transcriptomic clocks, and real‐time physiological monitoring, are now enabling accurate, minimally invasive assessment of circadian phase, opening new opportunities for personalized, time‐aware treatment planning [10]. These innovations mark a critical turning point, transforming chronotherapy from a theoretical ideal into a measurable, data‐driven discipline.

A clear distinction should be made between mechanistic plausibility and clinically validated benefit. In hematological malignancies, circadian regulation of hematopoiesis, immune function, DNA repair, drug metabolism, and tissue recovery provides a strong biological rationale for time‐aware treatment strategies. However, the available clinical evidence remains limited, heterogeneous, and largely retrospective. Therefore, chronotherapy in hematological oncology should currently be viewed as a promising investigational approach rather than an established therapeutic standard.

**FIGURE 1:**
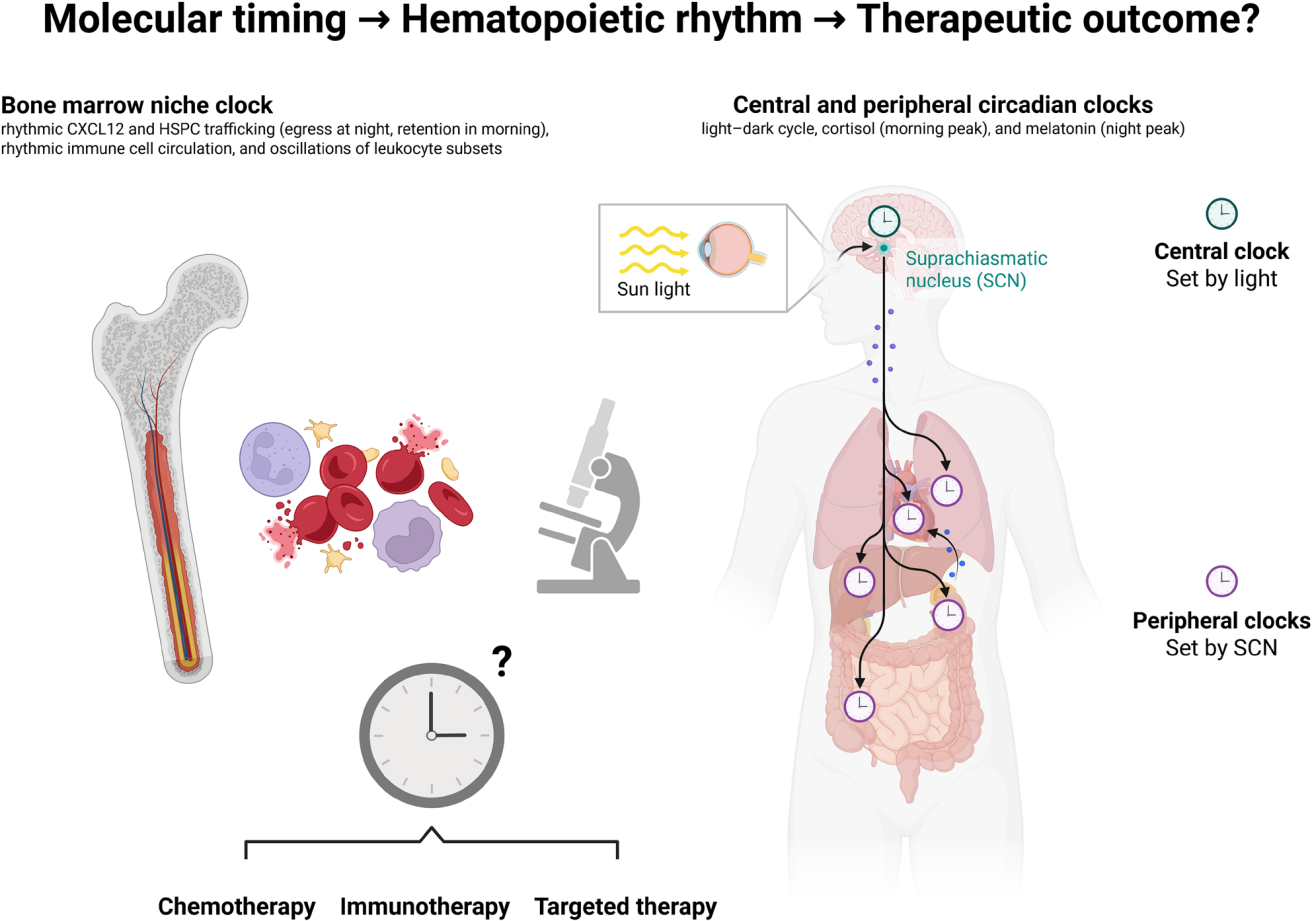
Conceptual framework linking central and peripheral circadian clocks to hematopoietic rhythms and time‐dependent therapeutic interventions in hematologic malignancies.

This review aims to provide a comprehensive synthesis of current knowledge linking circadian regulation to hematopoietic and malignant processes and evaluates the mechanistic, preclinical, and clinical evidence for chronotherapy in hematological malignancies. We begin by outlining the molecular and systemic architecture of circadian regulation, followed by its role in normal hematopoiesis and immune function. We then summarize lessons from chronotherapy in solid tumors, describe the current therapeutic landscape in hematology, and critically assess the emerging mechanistic, preclinical, and clinical evidence for circadian timing in leukemia, lymphoma, and myeloma. Finally, we identify practical challenges, potential solutions, and future directions, emphasizing that timing represents a critical yet overlooked axis of precision medicine. By reframing therapeutic optimisation through the lens of biological clock, chronotherapy may provide a novel investigational framework for improving efficacy, reducing toxicity, and individualizing care, provided that its clinical value is confirmed in prospective hematology‐specific trials (Figure 1).

## 2 Fundamentals of Circadian Biology

Life on Earth has evolved under 24‐h cycles of light and darkness, and nearly all organisms, from cyanobacteria to mammals, have developed endogenous clocks that anticipate and adapt to these environmental oscillations [11]. At the cellular level, circadian timing is generated by self‐sustained transcriptional‐translational feedback loops. The CLOCK‐BMAL1 transcriptional complex activates the expression of Period (PER1‐3) and Cryptochrome (CRY1‐2) genes, whose protein products subsequently repress CLOCK‐BMAL1 activity and thereby close the negative feedback loop. Additional regulatory loops involving REV‐ERBα/β and RORα/γ stabilize BMAL1 expression and fine‐tune the amplitude and phase of circadian oscillation. Through this molecular architecture, peripheral and central clocks generate near‐24‐h rhythms in thousands of clock‐controlled genes involved in metabolism, cell‐cycle control, DNA repair, oxidative stress responses, immune activity, and xenobiotic handling [12, 13, 14]. Beyond this molecular core, chromatin remodeling and post‐translational modifications, such as phosphorylation by casein kinase 1ε/δ, introduce further precision, modulating period length and feedback stability [15, 16].

As every cell in our bodies possesses an autonomous oscillator, systemic synchronization is essential. The coherence of these cellular clocks depends on entrainment by “zeitgebers”, time‐giving cues that align internal cycles with the external environment [6, 17]. In mammals, a master pacemaker located in the suprachiasmatic nucleus (SCN) of the hypothalamus synchronizes subsidiary clocks throughout the body, ensuring that physiological processes remain temporally coordinated [18]. The SCN receives direct photic input from retinal ganglion cells via the retino‐hypothalamic tract and translates these signals into rhythmic patterns of neuronal and hormonal output [19, 20]. Through sympathetic and parasympathetic neural pathways, as well as endocrine mediators such as cortisol and melatonin, the SCN entrains clocks in virtually every peripheral tissue [21, 22]. In addition, other inputs, such as feeding‐fasting cycles, body temperature, and hormonal rhythms, act as secondary zeitgebers that entrain peripheral tissues [23, 24, 25]. Together, these signals maintain phase coherence among organs while allowing adaptive flexibility; for example, the liver clock is particularly sensitive to feeding, whereas the adrenal and pineal clocks respond primarily to neural and hormonal input [26]. Disruption of this coupling, through shift work, jet lag, or irregular sleep patterns, desynchronizes cellular clocks and perturbs metabolic and immune homeostasis [27, 28]. Such desynchronization leads to metabolic inefficiency, elevated oxidative stress, and impaired tissue repair, establishing a physiological substrate for chronic disease [29, 30].

Circadian regulation thus extends beyond mere chronometry; it governs the timing of physiological reactions. Blood pressure, hormone secretion, mitochondrial respiration, xenobiotic metabolism, and immune activation all follow robust daily oscillations [31, 32, 33]. In health, these temporal programs optimize energy utilization and repair; in disease, their breakdown contributes to pathological states ranging from metabolic syndrome to cancer [34, 35]. One of the key systems regulated by circadian rhythms is the immune system [36, 37].

## 3 Circadian Regulation of Hematopoiesis and Immunity

The hematopoietic system is inherently rhythmic: proliferation, differentiation, and mobilization of stem and progenitor cells follow daily cycles coordinated by both intrinsic molecular clocks and extrinsic neuroendocrine cues. Within the bone marrow, sympathetic output from the SCN fluctuates across the day, releasing norepinephrine that acts on stromal β3‐adrenergic receptors to regulate CXCL12 expression and control hematopoietic stem and progenitor cell (HSPC) trafficking [38]. Downregulation of CXCL12 during the resting phase facilitates nocturnal egress of HSPCs [39]. Endocrine mediators such as cortisol and melatonin further refine this rhythm: cortisol peaks in the early morning, modulating CXCL12 transcription and leukocyte mobilization, while melatonin secreted during darkness supports HSPC maintenance by reducing oxidative stress and enhancing self‐renewal of quiescent cells [40, 41, 42, 43].

These coordinated influences align hematopoietic renewal with periods of metabolic recovery, ensuring temporal coherence between regenerative and systemic physiology. Clinically, stem‐cell collection and engraftment efficiency fluctuate with circadian phase; HSPCs harvested at specific times yield superior numbers and repopulation potential [9]. Similarly, sensitivity to cytotoxic stress and reparative capacity vary with cell‐cycle and redox oscillations, providing a mechanistic rationale for the time‐dependent modulation of therapy [41, 44].

Circadian control extends beyond stem‐cell dynamics to the behavior of mature blood cells. Neutrophils display striking rhythmicity: circulating numbers peak during the active phase under adrenergic influence, whereas nocturnal tissue migration is mediated by CXCR4 signaling and core clock‐dependent transcription. This rhythmic turnover maintains immune readiness while limiting vascular inflammation; its disruption enhances bacterial clearance but increases thrombo‐inflammatory risk [45, 46]. Lymphocyte subsets follow distinct temporal programs: cytotoxic T cells and natural‐killer (NK) cells peak during the day under epinephrine‐driven activation, whereas naïve and regulatory T cells accumulate at night in response to cortisol and melatonin. Sleep suppresses adrenergic tone, promoting T‐cell homing to lymphoid tissues and facilitating adaptive immune responses [47]. Monocytes and macrophages oscillate via clock‐regulated adhesion molecules and chemokine receptors, synchronizing inflammatory tone with metabolic rhythms and preventing excessive activation during wakefulness [48, 49]. Platelets also display diurnal variation in aggregation and granule release, contributing to the morning peak in thrombosis, while erythrocytes manifest intrinsic redox oscillations that modulate oxygen delivery and antioxidant capacity [50].

When these rhythmic patterns deteriorate through aging, metabolic stress, or chronic inflammation, hematopoietic and immune equilibrium shifts toward maladaptation, predisposing to chronic inflammatory states and disease vulnerability [51]. At the molecular level, chronic disruption of systemic cues such as light–dark cycles, feeding patterns, glucocorticoid pulses, and temperature rhythms desynchronises central and peripheral clocks, abolishes rhythmic leukocyte expression, and impairs hematopoietic balance [39]. In humans, misalignment between cortisol and melatonin rhythms disrupts clock‐gene oscillation in immune cells, reflecting molecular desynchrony that compromises tissue repair and recovery [52, 53].

Taken together, these findings demonstrate that hematopoiesis and immunity are not static processes but temporally orchestrated networks. Their rhythmic behavior provides both the biological rationale for chronotherapy and a mechanistic foundation for understanding why treatment timing can profoundly influence therapeutic outcomes.

## 4 Chronotherapy in Cancer Pharmacology

The concept of chronotherapy originated in solid‐tumor oncology, where daily oscillations in drug metabolism, cell‐cycle phase, and DNA repair capacity profoundly influence the balance between efficacy and toxicity. Every component of drug disposition, absorption, distribution, metabolism, and excretion, fluctuates across the day under circadian control [28, 54, 55]. Rhythmic variations in hepatic blood flow, enzyme expression, and renal clearance determine the pharmacokinetics of many chemotherapeutics, while oscillations in cell proliferation and apoptosis modulate pharmacodynamic sensitivity in both tumor and normal tissues. These diurnal variations underpin the principle that identical drug doses can yield markedly different outcomes depending on the biological time of administration.

Classical cytotoxic agents such as cisplatin, doxorubicin, 5‐fluorouracil (5‐FU), and oxaliplatin display clear time‐of‐day‐dependent differences in tolerability and therapeutic index. For instance, 5‐FU metabolism depends on dihydropyrimidine dehydrogenase (DPD), an enzyme whose activity peaks during the resting phase, thereby reducing systemic exposure when drugs are administered in synchrony with this rhythm [56]. Conversely, nocturnal dosing of oxaliplatin and irinotecan, agents metabolized by rhythmic hepatic carboxylesterases and cytidine deaminase, can minimize toxicity while maintaining efficacy [57, 58]. Such findings led to the development of chronomodulated infusion schedules, notably in colorectal and ovarian cancers, where timed delivery improved outcomes and reduced adverse events compared with conventional flat‐rate infusion [59].

Pharmacokinetic processes are not temporally uniform: absorption, distribution, metabolism, and elimination each exhibit 24‐h periodicity under circadian control, such that the time of administration materially influences systemic drug exposure. During flat‐rate 5‐FU infusion, dihydropyrimidine dehydrogenase activity and plasma drug concentrations oscillate inversely, the latter differing almost five‐fold across 24 h [60]. Analogous rhythms in metabolizing enzymes, transporters, hepatic blood flow, and renal clearance, together with circadian variation in target sensitivity, DNA repair, and immune competence, shift the efficacy‐toxicity balance, so that the tolerability of cytotoxic agents can vary two‐ to ten‐fold with dosing time [59, 61]. Population pharmacokinetic‐pharmacodynamic models incorporating clock‐driven parameters now allow this therapeutic window to be predicted and personalized [62].

Beyond pharmacokinetics, circadian timing also shapes tumor‐intrinsic biology through the core molecular clock described above. Clock‐controlled regulation of cell‐cycle progression, DNA repair, p53 stability, MYC transcriptional output, and homologous recombination efficiency positions biological time as a determinant of genomic integrity. Experimental models confirm that disruption of clock genes such as Per2 or Bmal1 accelerates tumor initiation, while restoring rhythmicity through timed feeding or light exposure slows tumor growth [13, 63]. Once established, however, tumors often exhibit intrinsic circadian oscillations that influence proliferation, metabolic flux, and therapeutic response. Wagner et al. [64] demonstrated that tumor growth rates vary predictably over 24 h. Tumor cells with impaired circadian machinery become metabolically inflexible and more sensitive to stress imposed at specific circadian phases. Moreover, desynchronisation between tumor and host rhythms may create exploitable temporal windows in which therapy delivered at phases of maximal tumor activity and minimal host susceptibility achieves superior efficacy [65].

Leveraging this vulnerability through timed therapy represents a form of biological targeting distinct from molecular inhibition. Modern experimental systems allow systematic dissection of these temporal effects. Using in vitro phenotyping pipelines, Lee et al. [4, 5] synchronized U2OS osteosarcoma cells with dexamethasone and tested more than 120 anticancer compounds across six circadian phases. Drug responses varied up to 30% depending on dosing time, even under identical concentrations. Likewise, Ector et al. [66] monitored luciferase‐tagged cell lines through live‐cell imaging and found consistent phase‐dependent differences in growth inhibition across multiple agents, indicating that circadian timing influences virtually every major class of chemotherapeutic compound. These high‐throughput screening studies establish a scalable framework for mapping circadian pharmacodynamics, an approach increasingly applicable to drug discovery pipelines.

Clinical studies reinforce these findings. Meta‐analyses across multiple solid tumors, including urothelial, esophageal, non‐small‐cell lung, melanoma, and renal‐cell carcinoma, demonstrated significantly improved overall survival when immune‐checkpoint inhibitors (ICIs) were delivered in the morning rather than later in the day [67]. Another systematic review of 21 studies confirmed this pattern across cancer types, though heterogeneity in timing definitions and circadian assessment methods highlights the need for standardization [68]. In advanced colorectal cancer, for example, chronomodulated FOLFOX regimens (5‐FU, leucovorin, and oxaliplatin) achieved comparable or improved response rates with markedly reduced mucosal, haematologic, and neurological toxicity compared with constant‐rate infusions [59, 69]. Similar findings have been reported in ovarian, head‐and‐neck, and lung cancers. However, inter‐individual differences in chronotype, sex, and hormonal status substantially influence optimal timing. For instance, female patients tend to experience peak toxicity several hours earlier than males, necessitating personalized timing adjustments [59].

Chronotherapy has been extended to targeted and immune‐based therapies. Tyrosine‐kinase inhibitors (TKIs), including erlotinib and sunitinib, display circadian variation in pharmacokinetics and efficacy due to rhythmic expression of metabolic enzymes and drug transporters [70, 71]. In murine models, everolimus, an mTOR inhibitor, produced greater tumor growth inhibition and prolonged survival when administered during periods of high mTOR activity [72].

The circadian clock also regulates multiple aspects of immune function directly relevant to cancer therapy, including T‐cell activation, dendritic‐cell migration, and cytokine release. These processes are central to the mechanisms of action of immunotherapies, particularly ICIs [68, 73]. During the morning hours, immune competence peaks, characterized by enhanced dendritic‐cell migration, heightened responsiveness of CD4^+^ and CD8^+^ T cells, and increased production of B lymphocytes [74, 75, 76]. Because ICIs depend on these antigen‐presentation and T‐cell activation pathways, treatments administered during this phase may elicit stronger antitumor immunity and fewer immune‐related toxicities, highlighting the clinical relevance of dosing time in immunotherapy. Preclinical evidence supports this concept in adoptive cell therapies. In murine melanoma models, chimeric antigen receptor (CAR) T‐cell infusions administered during the active phase achieved superior tumor control and more favorable cytokine profiles than those given during the rest phase [76]. These findings imply that aligning cellular immunotherapies with endogenous circadian rhythms could enhance antitumor potency. Whether such temporal optimisation can improve CAR‐T efficacy or mitigate cytokine‐release syndrome in patients with hematological malignancies remains an open yet promising question.

Together, these findings establish circadian timing as a fundamental variable in cancer pharmacology. The temporal architecture of drug metabolism, immune activation, and tumor biology provides both mechanistic insight and practical leverage. The success of chronotherapy in solid tumors sets a crucial precedent for its application in hematological malignancies, where cell proliferation, differentiation, and immune modulation are equally rhythmic but less studied. The next section explores how these principles extend to the haematologic domain and how chronotherapy may transform the treatment landscape of leukemias, lymphomas, and myelomas.

## 5 Current Landscape in Chemotherapy for Hematological Malignancies

Despite the molecular precision achieved with modern targeted agents, classical cytostatic agents remain the therapeutic backbone of haematologic oncology. Their continued use is not the product of historical inertia but reflects their unmatched ability to induce rapid cytoreduction in proliferative diseases and to serve as synergistic partners in combined‐modality regimens. Chemotherapy remains essential for achieving remission, controlling disease burden, and preparing patients for transplantation, even as targeted and immune‐based approaches redefine the therapeutic landscape.

The central rationale for cytostatic chemotherapy is its capacity to induce cytotoxicity in rapidly dividing malignant hematopoietic cells, activating intrinsic mechanisms of self‐destruction through the p53 pathway [77, 78]. This leads to a swift reduction in tumor burden, which is especially vital in high‐turnover tumors that may otherwise cause life‐threatening complications such as tumor‐lysis syndrome, hyperleukocytosis, or compression phenomena. Because classical agents lack selectivity for malignant over normal cells, their use is often constrained by hematopoietic and mucosal toxicities. Furthermore, impairment of p53 functionality through mutation or deletion diminishes their efficacy, underscoring the need for adjunctive or alternative strategies in genetically complex disease.

Chemotherapy continues to anchor first‐line regimens across virtually all haematologic malignancies. In acute myeloid leukemia (AML), induction therapy with the traditional 3 + 7 regimen, comprising an anthracycline and cytarabine, remains the standard of care for fit patients. This combination achieves rapid cytoreduction and high remission rates. The regimen may be augmented with targeted agents such as FLT3 inhibitors in FLT3‐mutated patients or gemtuzumab ozogamicin in favorable‐risk groups, and is typically followed by high‐dose cytarabine consolidation and allogeneic stem‐cell transplantation. In aggressive non‐Hodgkin lymphomas, polychemotherapy regimens such as R‐CHOP (rituximab, cyclophosphamide, hydroxydaunorubicin, vincristine, prednisone) and its derivatives remain the standard of care. In Hodgkin lymphoma, first‐line treatment still relies on classical cytostatics used in regimens like ABVD, BV‐AVD, BEACOPP, or BrECADD (containing brentuximab vedotin, doxorubicin, vincristine, dacarbazine, etoposide, bleomycin, cyclophosphamide, procarbazine) which form the basis for curative therapy. In multiple myeloma, melphalan continues to play a central role, serving as the backbone of some combination regimens for transplant‐ineligible patients and as a myeloablative conditioning agent in autologous transplantation. Among Philadelphia‐negative myeloproliferative neoplasms, hydroxyurea remains the first‐line cytoreductive agent of choice for controlling blood counts and reducing thrombotic risk. It is also used for rapid cytoreduction in AML patients with hyperleukocytosis.

Targeted therapies have revolutionized the management of hematological malignancies by selectively inhibiting oncogenic drivers or surface antigens, achieving high response rates with generally lower toxicity when added to or replacing conventional chemotherapy. Landmark examples include imatinib, the first BCR::ABL tyrosine‐kinase inhibitor that transformed chronic myeloid leukemia (CML) from a fatal disease to a manageable condition, and rituximab, the anti‐CD20 monoclonal antibody that drastically improved outcomes in B‐cell lymphomas. These agents exemplify the precision medicine paradigm by targeting molecular abnormalities unique to the tumor. However, limitations persist. Resistance mutations, clonal evolution, and drug‐specific toxicities frequently undermine long‐term control, while high costs restrict accessibility in many regions [79]. Moreover, most targeted therapies still depend on chemotherapy backbones for optimal efficacy or as bridging therapy before cellular or immune‐based interventions. For example, FLT3‐ or IDH‐inhibitors in AML, and BCL2‐ or BTK‐inhibitors in lymphoid malignancies, are often combined with or sequenced after cytotoxic regimens. Similarly, cytostatic agents remain integral to conditioning regimens for both autologous and allogeneic stem‐cell transplantation, where controlled myeloablation is necessary for engraftment.

This contemporary therapeutic landscape sets the stage for exploring how chronotherapy principles might refine current practice. Classical cytostatic and targeted agents alike interact with biological processes, DNA replication, metabolism, apoptosis that are under circadian control. The proliferative rhythms of malignant clones, the oscillation of drug‐metabolizing enzymes, and the daily variation in immune and hematopoietic regeneration create potential temporal windows in which treatment may be most effective or least toxic. Understanding this baseline, the pharmacological and clinical architecture of standard therapy, is essential for contextualizing the chronotherapeutic strategies discussed in subsequent sections.

## 6 Chronotherapy in Hematological Malignancies: Mechanistic and Preclinical Studies

**FIGURE 2:**
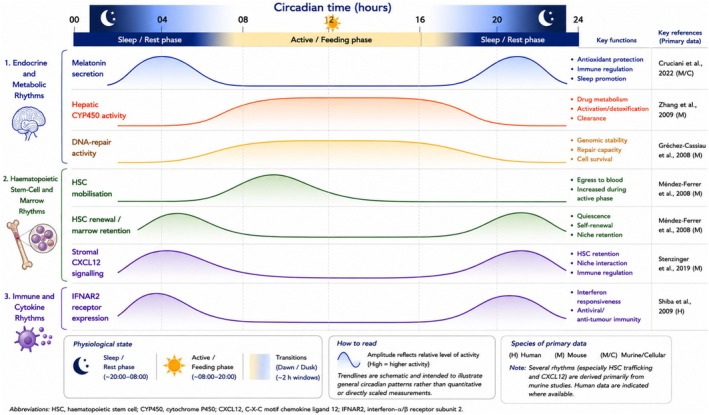
Circadian regulation of biological processes relevant to chronotherapy in hematological malignancies.

Hematological malignancies offer a unique framework for studying chronotherapy because the hematopoietic system itself is inherently circadian. Oscillations in proliferation, differentiation, and trafficking of hematopoietic and immune cells create dynamic landscapes of vulnerability and resilience that may strongly influence treatment outcomes. Emerging mechanistic and preclinical studies reveal that the molecular clock not only regulates normal hematopoiesis but also shapes malignant transformation, therapeutic sensitivity, and drug toxicity (Figure 2 and Table 1).

**TABLE 1:** Preclinical and clinical evidence supporting chronotherapy in hematological malignancies.

Evidence category	Disease/model	Intervention/mechanism	Timing comparison/circadian finding	Main outcome/translational implication	References
Preclinical	Murine hematological malignancy models	Circadian disruption	Chronic circadian misalignment vs. normal rhythm	Accelerated tumor initiation, genomic instability, and pro‐inflammatory tumor microenvironment	[63, 80]
	Leukemia and lymphoma models	Dysregulation of CLOCK, BMAL1, PER, CRY, REV‐ERB, and ROR pathways	Altered circadian clock‐gene expression	Promoted proliferation, altered metabolism, apoptosis resistance, and chemoresistance	[2, 81]
	Leukemia models	BMAL1 deletion/PER2 regulation	Circadian‐clock perturbation	BMAL1 deletion accelerated leukemogenesis; PER2 maintained genomic stability through p53‐mediated DNA repair	[82]
	Leukemia stem‐cell models	CLOCK or BMAL1 disruption	Circadian‐clock inhibition	Depletion of leukemia stem cells with limited effects on normal HSCs, suggesting therapeutic selectivity	[83, 84]
	Leukemia microenvironment models	Stromal‐clock dysregulation	Altered CXCL12 rhythmicity and immune timing	Increased oxidative stress, altered immune surveillance, and possible treatment resistance	[12]
	Chronic myeloid leukemia and myeloproliferative‐disorder models	Interferon‐α (IFN‐α)	IFNAR2 expression peaks nocturnally	Suggested improved response and reduced toxicity with night‐time administration	[85]
	Hematopoietic stem‐cell biology	Cytotoxic chemotherapy	Administration during HSC quiescence	Potential reduction in immunosuppression and bone marrow toxicity	[86]
	Hepatic chronopharmacology	Cytochrome P450 rhythmicity	Circadian oscillation in hepatic drug metabolism	Potential optimisation of drug activation, clearance, efficacy, and toxicity	[87]
Clinical	Pediatric acute lymphoblastic leukemia (ALL)	Maintenance chemotherapy	Evening vs. morning administration	Earlier studies suggested improved disease‐free survival with evening dosing; later study failed to confirm association	[88, 89, 90]
	Diffuse large B‐cell lymphoma	R‐CHOP chemotherapy	Afternoon vs. morning administration	In women, afternoon dosing improved progression‐free survival and overall survival and reduced infection and febrile neutropenia	[91]
	Anthracycline‐treated hematological malignancies	Anthracycline‐based chemotherapy	Later‐day vs. earlier‐day administration	Increased risk of cancer therapy‐related cardiac dysfunction and heart failure with later administration	[69]
	Allogeneic hematopoietic stem‐cell transplantation	Donor stem‐cell infusion timing	Before 2:00 p.m. vs. later infusion	Earlier infusion reduced grade II–IV and grade III–IV acute graft‐versus‐host disease and lowered transplant‐related mortality	[92]
	Stage IV Hodgkin lymphoma	Chronotherapy‐adjusted chemotherapy schedules	Circadian‐aligned vs. standard scheduling	Higher melatonin levels, enhanced liver‐enzyme activity, improved quality of life, and superior clinical outcomes	[93]

Epidemiological and experimental studies consistently associate chronic circadian misalignment with heightened risk of haematologic malignancy and accelerated tumor progression [80]. Mechanistically, temporal disruption undermines DNA repair by uncoupling NAD^+^ and PARP‐mediated pathways, suppressing p21^WAF/CIP1 and promoting genomic instability [94]. Murine models confirm that persistent circadian disturbance accelerates tumor initiation and remodels the immune microenvironment toward a pro‐inflammatory, pro‐tumorigenic state [63, 80].

The circadian clock functions as both a guardian of hematopoietic homeostasis and a potential vulnerability in hematological cancers. Rather than repeating the molecular architecture outlined above, it is important here to emphasize its pathological distortion: clock‐gene dysregulation in leukemia and lymphoma has been associated with uncontrolled proliferation, altered metabolism, impaired apoptosis, and chemoresistance [2, 81]. Specific perturbations within this network may influence malignant behavior; for example, BMAL1 loss has been linked to accelerated leukemogenesis through deregulation of MYC and E2F, whereas PER2 appears to support genomic stability through p53‐mediated DNA repair [82]. In leukemia models, clock reprogramming may therefore contribute to therapeutic resistance by uncoupling cell‐cycle progression from DNA repair and enabling malignant cells to survive genotoxic stress [13, 55, 95].

Functionally, leukemia stem cells appear dependent on intact circadian machinery: disruption of CLOCK or BMAL1 depletes these cells and promotes differentiation, with minimal effects on normal hematopoietic stem cells [83]. These findings suggest that circadian dysregulation is not merely a bystander effect but a driver of malignant persistence and therapeutic resistance. The same molecular circuitry that coordinates physiological renewal in health becomes distorted in malignant clones, creating asynchronous proliferation and temporal heterogeneity in drug sensitivity [12, 83].

Chronobiological analyses also reveal that circadian disruption reshapes the tumor microenvironment. In leukemia models, aberrant clock‐gene expression affects stromal CXCL12 rhythms, alters immune surveillance, and increases oxidative stress, all of which may foster leukemic expansion. Temporal misalignment between malignant and stromal clocks could therefore underpin treatment resistance, particularly to agents that rely on proliferative synchrony or immune‐mediated cytotoxicity.

Multiple preclinical studies have tested the impact of treatment timing on antitumour efficacy in hematological malignancies and related models. Evidence consistently indicates that aligning therapy with circadian phase can enhance efficacy and reduce toxicity [96]. One of the earliest demonstrations of circadian control in hematology came from studies on interferon‐α (IFN‐α), a targeted therapy used in chronic myeloid leukemia (CML) and other myeloproliferative disorders. IFN‐α administration was shown to alter the expression of core clock genes and modulate light‐induced gene activation in the SCN. Importantly, expression of the interferon‐α/β receptor subunit IFNAR2 in peripheral blood mononuclear cells peaks at night, suggesting that nocturnal administration could improve therapeutic response and minimize adverse effects [85]. Furthermore, because bone marrow proliferation and DNA synthesis peak at specific circadian phases, administering cytotoxic agents when healthy HSCs are quiescent could mitigate immunosuppression [86].

Collectively, these mechanistic and preclinical insights lay the groundwork for translating chronotherapy into hematological oncology. They demonstrate that both malignant and normal hematopoietic compartments exhibit clock‐dependent vulnerabilities that can, in theory, be harnessed to enhance efficacy and safety.

## 7 Chronotherapy in Hematological Malignancies: Clinical Experiences

The clinical evidence for chronotherapy in hematological malignancies should be interpreted cautiously. Although mechanistic and preclinical findings provide a strong rationale for time‐dependent treatment effects, available clinical data are still limited by retrospective design, small sample sizes, heterogeneous regimens, and inconsistent definitions of treatment timing. Accordingly, the studies discussed below should be regarded as hypothesis‐generating rather than definitive evidence of clinical benefit.

While the biological rationale for circadian modulation in hematology is strong, clinical evidence remains limited and heterogeneous. Most available studies are retrospective, involve small cohorts, or use regimens that pre‐date the era of targeted therapy. Nonetheless, as summarized in Table 1, accumulating evidence from mechanistic, preclinical, and early clinical studies suggests that treatment timing may influence therapeutic efficacy, toxicity, immune response, and transplantation outcomes across multiple hematological malignancies.

The first clinical observations linking circadian timing with outcome in hematological cancers date back to pediatric acute lymphoblastic leukemia. Two early reports on the same cohort described that children receiving maintenance chemotherapy in the evening had more favorable disease‐free survival compared with those treated in the morning [88, 89]. These findings suggested that the timing of drug administration might modulate relapse risk, perhaps through circadian variation in drug metabolism or bone marrow recovery. However, subsequent work by Clemmensen et al. [90] failed to confirm this association, illustrating the high variability that has continued to characterize chronotherapy trials in hematology. Differences in treatment era, dosing schedules, and circadian assessment methods may have contributed to the inconsistent results.

More recent analyses have focused on anthracycline‐containing regimens, particularly R‐CHOP in diffuse large B‐cell lymphoma. A retrospective study by Kim et al. [91] suggested that treatment timing may influence survival and hematological toxicity in a sex‐specific manner, with afternoon administration appearing more favorable in female patients. However, this observation should not be interpreted as a general recommendation for later‐day administration of anthracycline‐containing therapy. In contrast, Printezi et al. [69] reported that anthracycline‐associated cardiotoxicity may follow an opposite temporal pattern, with lower risk observed when treatment was administered earlier in the day. These apparently divergent findings indicate that chronotherapy effects are likely endpoint‐specific, with hematological tolerance and organ‐specific toxicity requiring separate evaluation.

Chronotherapy principles may also apply to transplantation. Allogeneic hematopoietic stem‐cell transplantation is a curative therapy for many hematological disorders but is frequently complicated by immune‐mediated toxicities such as acute graft‐versus‐host disease. A prospective cohort study by Hou et al. [92] demonstrated that the time of donor‐cell infusion significantly influenced aGVHD risk. Patients receiving stem‐cell infusions before 2:00 p.m. had a cumulative incidence of grade II–IV aGVHD of 20.6%, compared with 38.3% in those infused later in the day (*p* = 0.009). Severe grade III–IV aGVHD occurred in only 9.3% of the early‐infusion group versus 27.1% of the late group (*p* < 0.001). Multivariate analysis confirmed later infusion time as an independent predictor of severe aGVHD (HR = 1.16, 95% CI: 1.05–1.27, *p* = 0.003). Moreover, early infusions were associated with lower 3‐year transplant‐related mortality (13.8% vs. 24.8%, *p* = 0.041). Mechanistically, these benefits were attributed to reduced IL‐1α levels and dampened T‐cell activation when transplantation was performed earlier in the day, a host‐circadian effect mediated primarily by the recipient's immune rhythm.

The circadian control of hepatic metabolism and endocrine function provides another mechanistic rationale for timing therapy in hematology. Liver enzymes involved in drug metabolism, including cytochrome P450 isoforms, exhibit robust circadian patterns of activity [87]. Synchronizing chemotherapy with hepatic enzymatic rhythms may optimize drug activation or clearance, improving efficacy and reducing toxicity [2]. An illustrative example comes from patients with stage IV Hodgkin lymphoma treated using chronotherapy‐adjusted schedules. Those receiving chemotherapy aligned with their natural circadian rhythm exhibited higher melatonin levels, enhanced liver‐enzyme activity, and improved quality of life compared with standard regimens [93]. These patients also showed more favorable clinical outcomes, although these findings should be interpreted as preliminary and hypothesis‐generating until confirmed in larger prospective studies. The favorable effects were attributed, at least in part, to melatonin's antiproliferative, pro‐apoptotic, and antioxidant actions.

Although findings across studies vary, several common themes emerge. Time of administration may matter; measurable differences in outcomes between morning and afternoon dosing have been observed across multiple modalities: chemotherapy, immunotherapy, and stem‐cell transplantation. Host factors such as sex, hormonal status, and immune rhythmicity interact with timing to modulate therapeutic response, explaining apparent inconsistencies between studies. Chronotherapy may separate efficacy from toxicity; afternoon R‐CHOP improved haematologic tolerance in women, yet the same timing increased cardiotoxicity risk with anthracyclines, indicating that each agent and outcome variable requires specific optimisation. Endogenous hormonal and metabolic cycles modulate drug response, as circadian regulation of melatonin, cortisol, and hepatic metabolism contributes to differences in drug handling and oxidative stress response.

Overall, while the evidence base remains limited, these observations suggest that circadian timing may exert clinically relevant effects, but the strength of evidence remains insufficient for routine clinical implementation. The convergence of mechanistic plausibility, preliminary clinical data, and emerging technologies now positions chronotherapy as a testable dimension of precision hematology that requires prospective validation before implementation.

To consolidate the available evidence across specific agents and disease settings, Table 1 summarizes the key clinical, observational, and translational studies evaluating chronotherapy in hematological malignancies, spanning pediatric and adult leukemia, aggressive lymphoma, chronic lymphocytic leukemia, and stem‐cell transplantation. This overview highlights both the breadth and the heterogeneity of the existing literature, ranging from early retrospective observations in pediatric ALL to more recent cohort analyses in DLBCL and allogeneic transplantation.

Critically, the magnitude of timing‐associated effects observed across these studies is not trivial. In the DLBCL cohort reported by Kim et al. [91], afternoon administration of R‐CHOP—a regimen combining cyclophosphamide, doxorubicin, vincristine, and prednisone with rituximab—was associated with a 12.5‐fold lower mortality rate in female patients (2% vs. 25%) and a 2.8‐fold reduction in 5‐year cancer recurrence (13% vs. 37%), with significantly improved progression‐free survival (HR 0.357, *p* = 0.033) and overall survival (HR 0.141, *p* = 0.032). These effect sizes are clinically substantial and comparable to those achieved by established pharmacological interventions in the same disease. The haematologic tolerability advantage of afternoon dosing was attributed to diurnal fluctuations in circulating leukocyte counts, which are more pronounced in women, rendering them particularly vulnerable to morning‐administered myelosuppressive agents such as cyclophosphamide and doxorubicin. Conversely, anthracycline cardiotoxicity appears to follow the opposite temporal pattern: a large retrospective analysis by Printezi et al. [69] demonstrated that morning administration of anthracyclines was associated with a significantly lower risk of cancer therapy‐related cardiac dysfunction, a finding consistent across sex, age, and cumulative dose. This divergence between efficacy and toxicity optima for the same agent underscores the complexity of timing optimisation and the need for outcome‐specific scheduling strategies.

In the transplantation setting, timing effects of comparable magnitude have been reported for stem‐cell infusion. Hou et al. [92] demonstrated that patients receiving allogeneic hematopoietic stem‐cell infusions before 2:00 p.m. had a cumulative incidence of grade II–IV acute graft‐versus‐host disease of 20.6%, compared with 38.3% in those infused later in the day (*p* = 0.009), with severe grade III–IV aGVHD occurring in only 9.3% versus 27.1% of patients (*p* < 0.001). Transplant‐related mortality at 3 years was also significantly lower in the early‐infusion group (13.8% vs. 24.8%, *p* = 0.041). These differences, driven by circadian variation in recipient immune activation and IL‐1α levels, represent a clinically meaningful reduction in some of the most serious complications of allogeneic transplantation—achievable without any change in conditioning regimen, donor selection, or pharmacological prophylaxis.

Taken together, the available evidence suggests that circadian timing exerts effects of clinically meaningful magnitude across multiple drug classes and disease settings in hematological oncology. The challenge now lies in translating these retrospective and pilot data into prospective, randomized trials designed to define optimal schedules for specific agents and patient subgroups, with particular attention to the interaction between sex, chronotype, and drug‐specific toxicity profiles.

The current evidence should therefore be interpreted across three levels. First, mechanistic and preclinical studies provide biological plausibility by showing that circadian clocks regulate hematopoietic proliferation, immune activation, DNA repair, oxidative stress responses, and drug metabolism. Second, retrospective and small clinical studies suggest that treatment timing may be associated with differences in toxicity or outcome in selected settings. Third, prospective randomized trials with validated circadian phase assessment are still required to establish whether these associations translate into clinically actionable benefit.

## 8 Challenges and Practical Considerations

Despite compelling mechanistic and early clinical evidence, the translation of chronotherapy into routine hematological practice remains limited. The primary challenge lies in the multidimensional nature of circadian variability, which extends beyond drug pharmacokinetics to encompass patient chronotype, disease biology, and healthcare logistics. Each of these factors introduces variability that complicates the definition of a single “optimal” treatment time.

Inter‐individual variability is perhaps the most substantial obstacle. Circadian phase can differ by several hours between individuals due to genetic polymorphisms, age, sex, and environmental influences [97]. Disease‐related factors further compound this heterogeneity. Cancer itself can reprogram peripheral clocks, leading to phase shifts in metabolism, hormone secretion, and immune activity that obscure the usual day–night pattern [98]. In hematological malignancies, such desynchronisation may be particularly pronounced, as the malignant clone and normal hematopoietic cells share the same microenvironment and compete for circadian regulatory inputs. In addition, frequent hospitalization, sleep fragmentation, and nocturnal nursing interventions further disrupt endogenous rhythms, effectively “flattening” circadian signals and complicating the identification of a stable biological timing window.

The design of clinical trials also presents specific challenges. Most hematology regimens are delivered as combination therapies, each drug with distinct pharmacokinetics and mechanisms of action. Establishing the optimal circadian schedule for a multi‐agent regimen such as R‐CHOP or 3 + 7 is vastly more complex than for single‐agent chronotherapy. Moreover, endpoints differ, while timing may influence acute toxicity within hours or days; effects on progression‐free or overall survival may require years to discern. Randomized chronotherapy trials must therefore be large, long, and precisely controlled for confounders such as baseline performance status, sex, and comorbidities.

Practical implementation within hospitals adds another layer of difficulty. Oncology units typically follow fixed daytime operating hours, and drug preparation, pharmacy, and nursing schedules are rarely optimized for variable dosing times. Administering chemotherapy late at night or early in the morning is logistically demanding, requiring staff availability, modified workflows, and potentially higher costs. Even if clinical benefit is demonstrated, adoption will depend on institutional willingness to restructure care delivery around circadian principles. Such constraints explain why chronotherapy, despite its scientific robustness, has not yet achieved widespread uptake in oncology practice. In particular, inpatient chemotherapy logistics often rely on centralized pharmacy compounding and batch preparation systems that are not compatible with flexible or patient‐specific timing, while infusion scheduling is constrained by chair availability, nursing workload distribution, and safety protocols that prioritize operational efficiency over temporal optimisation.

An additional complexity arises from the dual nature of hematological malignancies themselves. Unlike solid tumors, leukemias and lymphomas involve the very system that governs circadian regulation of immunity and hematopoiesis. Malignant clones and normal progenitors coexist in the same compartment, often sharing transcriptional and metabolic programs. This overlap complicates efforts to identify time windows that selectively target tumor cells while sparing normal marrow. Furthermore, disease‐induced inflammation and cytokine release can blunt circadian amplitude, diminishing the temporal separation between healthy and malignant tissues that chronotherapy seeks to exploit [63]. In aggressive or relapsed disease, the circadian system may be too disrupted to serve as a reliable guide for therapy scheduling.

Drug‐specific and toxicity‐related trade‐offs further limit clinical generalization. As discussed above, the same administration window may be favorable for one endpoint but unfavorable for another, particularly when hematological toxicity and organ‐specific toxicity are considered separately. These inconsistencies underline the need to distinguish timing for efficacy from timing for safety, which may not coincide. Optimisation may therefore require multi‐parameter modeling of drug kinetics, target engagement, patient chronotype, and organ‐specific vulnerability rather than reliance on fixed universal timepoints.

To address these limitations, an integrated approach combining patient‐specific circadian profiling, pharmacogenomic stratification, and adaptive scheduling algorithms is needed. Emerging technologies such as wearable sensors, AI‐based rhythm analysis, and transcriptomic phase‐prediction models [10] may soon allow personalized chronotherapy that adjusts in real time to each patient's internal clock. Incorporating circadian parameters into electronic medical records and infusion software could further facilitate implementation. Ultimately, the future of chronotherapy in hematology will depend not only on biological validation but also on infrastructural innovation—linking laboratory chronobiology to the realities of clinical care. However, feasibility must also be critically considered in high‐acuity settings, where critically ill patients (e.g., those in intensive care units or with febrile neutropenia) often require continuous monitoring, vasopressor support, or urgent unscheduled interventions, making strict adherence to circadian treatment windows impractical or unsafe. In such contexts, patient stability and clinical urgency necessarily supersede temporal optimisation, limiting the applicability of chronotherapeutic protocols.

In parallel, standardization of reporting practices will be essential. Few clinical trials currently document the time of day at which therapy was administered, hindering meta‐analyses and reproducibility. Simple documentation of administration times, rest–activity patterns, and light exposure could transform retrospective datasets into chronobiological resources. Establishing a consensus framework, similar to the CONSORT guidelines, specifically for chronotherapy studies would ensure methodological rigor and comparability across trials.

In summary, while the principles of chronotherapy are well established, their translation to hematological oncology faces biological, technical, and organizational barriers. Circadian variability among patients, disruption of rhythms by the disease itself, logistical constraints of hospital practice, and the absence of validated biomarkers collectively slow progress. Nevertheless, the accumulating evidence that timing can influence both efficacy and toxicity warrants continued investigation. The next step for the field is to move from proof‐of‐concept studies toward integrated, technology‐supported clinical trials that test personalized scheduling strategies grounded in each patient's biological time.

## 9 Future Directions and Emerging Technologies

The convergence of circadian biology, precision oncology, and digital health technologies is redefining what chronotherapy can achieve in hematology. Although the discipline remains at a formative stage, several technological and conceptual advances are now making it possible to measure, model, and manipulate the circadian system in real‐world clinical contexts. These developments promise to transform chronotherapy from a theoretical construct into a data‐driven and patient‐specific approach.

One major area of progress is the development of robust circadian biomarkers. The traditional marker, dim‐light melatonin onset (DLMO), provides an accurate reference of internal phase but is impractical in most clinical settings [99]. Recent transcriptomic and metabolomic clocks now offer a scalable alternative. Algorithms trained on genome‐wide expression data can predict circadian phase from a single blood draw or even from routinely collected clinical samples [100]. These approaches have been validated against DLMO with an accuracy of within one hour and could, in principle, be embedded into oncology workflows. Parallel advances in wearable biosensors, capturing rest–activity cycles, skin temperature, and heart‐rate variability, enable continuous assessment of circadian phase in ambulatory patients [101]. Integrating such multimodal data streams may soon permit dynamic circadian profiling that reflects the combined effects of disease, therapy, and lifestyle.

Artificial intelligence and computational modeling will play an essential role in this transition. Machine‐learning frameworks capable of integrating pharmacokinetics, pharmacodynamics, and circadian biology can identify temporal windows of maximal efficacy and minimal toxicity for complex regimens. Early proof‐of‐concept studies have demonstrated that neural networks trained on pharmacological and physiological data can predict individualized dosing times for agents such as oxaliplatin and doxorubicin [10]. Extending these models to hematological malignancies would allow clinicians to simulate how variations in clock phase, hormone profiles, or bone marrow cycling alter drug exposure and treatment response. In parallel, in silico chronopharmacology platforms could guide the design of adaptive clinical trials, where dosing time is dynamically adjusted based on each patient's evolving circadian parameters.

Emerging experimental systems are also poised to deepen our mechanistic understanding. Ex vivo hematopoietic cultures and patient‐derived xenografts can now be entrained and sampled across circadian cycles to characterize how leukemia stem cells, stromal niches, and immune populations respond to temporal cues. Coupling these systems with single‐cell transcriptomics and proteomics could reveal phase‐dependent vulnerabilities that are obscured in time‐averaged assays. Synthetic biology adds another dimension: clock‐responsive promoters such as E‐box or REV‐ERB elements can be used to engineer cells or drug‐delivery vehicles that release therapeutic payloads in synchrony with endogenous rhythms [102, 103]. Such constructs offer the possibility of self‐regulating therapies—biological systems that sense time and act accordingly.

Taken together, these emerging technologies provide an increasingly sophisticated foundation for future circadian‐guided therapeutic strategies. Continued integration of chronobiology with biomarker science, computational modeling, and digital health technologies may ultimately enable more personalized and temporally optimized approaches to cancer treatment.

## 10 Regulatory and Translational Frameworks for Chronotherapy in Hematological Malignancies

As the above sections have shown, chronotherapy in cancer faces a unique translational challenge because circadian phase is shaped by chronotype, sex, hormonal status, sleep behavior, environmental cues, inflammation, and even the treatment itself. This complexity is particularly relevant in hematological malignancies, which directly involve the highly circadian‐regulated hematopoietic and immune systems. An important step toward overcoming this challenge will be the establishment of more systematic and harmonized methodological and reporting standards. At present, most studies on cancer interventions do not routinely document administration time, patients' rest‐activity patterns, chronotype, or environmental circadian modifiers such as light exposure, feeding schedules, corticosteroid use, and shift work history. In the future, it may be beneficial to develop standardized chronobiological reporting frameworks, analogous to CONSORT‐ or SPIRIT‐style extensions [104], for temporal medicine. Such frameworks could provide structured guidance for reporting treatment‐administration times, circadian biomarkers, chronotype assessment, environmental zeitgebers, and time‐of‐day‐stratified efficacy and toxicity analyses.

Another important consideration for clinical implementation is the development of practical and standardized methods for assessing circadian phase in individual patients [105]. Digital infrastructure and clinical workflow innovation will be equally critical. Hospital information systems could incorporate circadian modules that automatically propose dosing schedules based on patients' biological clocks, while infusion pumps and oral dosing apps could be programmed to deliver medication at optimal times. For outpatients, mobile platforms integrating wearable data, medication reminders, and symptom diaries could maintain circadian alignment throughout long treatment courses. These digital tools not only facilitate implementation but also generate high‐resolution data to refine predictive models continually. However, prior to widespread adoption, these technologies will require rigorous analytical and clinical validation through regulatory frameworks analogous to those used for companion diagnostics, digital biomarkers, and software‐as‐a‐medical‐device (SaMD) platforms [106, 107].

As the field evolves, chronopharmacology may become increasingly integrated into drug development. During preclinical and translational research, experimental models, such as cell‐culture systems, ex vivo hematopoietic models, and animal studies, can be used to characterize the chronobiology of candidate therapies, including circadian variation in pharmacokinetics, pharmacodynamics, target engagement, efficacy, and toxicity. Such studies could identify temporal vulnerabilities in both malignant and normal hematopoietic compartments and establish mechanistic rationale before clinical translation.

Subsequent translational efforts could incorporate routine prospective recording of treatment administration times and circadian metadata within existing oncology trials, thereby generating large chronobiologically informative datasets with minimal disruption to clinical workflows. This could be followed by biomarker‐validation and feasibility studies evaluating transcriptomic clocks, wearable‐derived phase estimators, and hormonal markers. Phase II studies could then assess whether circadian‐guided dosing improves pharmacodynamic responses, toxicity profiles, or biomarker‐defined endpoints before progressing to multicenter Phase III randomized trials powered for survival, toxicity reduction, quality‐of‐life improvement, or healthcare‐utilization outcomes.

Importantly, these studies should distinguish between chronoefficacy and chronotoxicity, as the optimal circadian window for tumor control may not coincide with that for minimizing adverse effects such as cardiotoxicity, myelosuppression, cytokine‐release syndrome, or immune‐related toxicity. This challenge is likely to become more complex in multi‐agent regimens, where individual drugs may possess distinct and potentially conflicting chronoefficacy and chronotoxicity profiles. Adaptive trial designs integrating circadian biomarkers, wearable monitoring, and AI‐assisted scheduling may help address these complexities [108]. Successful clinical implementation will also likely depend on infrastructural and organizational adaptation within healthcare systems. Over time, electronic medical records, infusion scheduling platforms, pharmacy systems, and mobile health technologies may begin to incorporate circadian‐aware functionalities capable of integrating biological time information into routine treatment planning.

Finally, future regulatory and governance frameworks will need to address ethical and societal issues unique to chronotherapy. Continuous circadian monitoring generates highly detailed behavioral and physiological data, raising concerns regarding privacy, informed consent, data ownership, and algorithmic transparency [109]. Equitable access will also be important, particularly as chronotherapy increasingly relies on wearable monitoring devices, AI‐assisted scheduling systems, and digitally integrated healthcare infrastructure that may not be equally available across healthcare settings [110]. Regulatory agencies and funding bodies may therefore need to support interoperable circadian‐data standards, collaborative research networks, and clinically interpretable AI platforms while ensuring that temporally personalized medicine remains accessible beyond technologically advanced centers.

Taken together, the future development of chronotherapy in hematological malignancies will depend not only on biological discovery, but also on coordinated advances in clinical methodology, biomarker validation, digital infrastructure, regulatory science, and healthcare implementation. Although substantial challenges remain, the convergence of chronobiology, precision medicine, wearable technologies, and AI‐assisted clinical systems is steadily transforming chronotherapy from a largely experimental concept into a potentially actionable therapeutic framework. Incorporating temporal precision as a standard dimension of treatment could inform future models of personalized supportive and antineoplastic care in hematological oncology, if validated in prospective trials.

## 11 Conclusions

Chronotherapy in hematological malignancies is supported by a coherent mechanistic framework and accumulating preclinical and early clinical evidence indicating that circadian timing can modulate drug efficacy, toxicity, immune function, and transplantation outcomes. However, the current evidence base remains largely heterogeneous, retrospective, and insufficient to support routine clinical implementation. Key limitations include substantial inter‐individual and disease‐related variability in circadian phase, frequent circadian disruption in patients due to malignancy, hospitalization, and treatment‐related factors, and the lack of validated, standardized biomarkers of internal biological time applicable in routine oncology practice. Further constraints arise from the complexity of multi‐agent chemotherapy regimens, potential dissociation between optimal timing for efficacy and toxicity endpoints, and the logistical and workforce demands of delivering time‐specific therapy within conventional hospital systems. In addition, most existing studies are underpowered, use inconsistent definitions of “time of day,” and lack prospective randomized designs controlling for key confounders such as sex, chronotype, and comorbidity. Robust translation into clinical practice will therefore require well‐designed prospective trials, harmonized chronotherapy reporting standards, validated circadian biomarkers, and integration of adaptive, technology‐enabled scheduling systems before chronotherapy can be considered an evidence‐based component of routine hematological oncology care.

## Author Contributions

**Marko Lucijanić:** conceptualization, investigation, writing – original draft, writing – review and editing. **Bruna Perkov‐Stipičin:** investigation, writing – original draft, writing – review and editing. **Yun Wah Lam:** conceptualization, investigation, writing – original draft, writing – review and editing. **Rossana Roncato:** investigation, writing – original draft, writing – review and editing, visualization. **Almir Fajkić:** conceptualization, investigation, writing – original draft, writing – review and editing, visualization. **Marko Skelin:** conceptualization, investigation, writing – original draft, writing – review and editing. **Ivan Krečak:** conceptualization, investigation, writing – original draft, writing – review and editing. **Andrej Belančić:** conceptualization, investigation, writing – original draft, writing – review and editing, project administration, visualization.

## Funding

The authors have nothing to report.

## Consent

The authors have nothing to report.

## Conflicts of Interest

The authors declare no conflicts of interest.

## Data Availability

No new data was generated. Additional information is available upon reasonable request sent to the corresponding author.
